# First person – Iris Sanou and Mathangi Lakshmipathi

**DOI:** 10.1242/bio.062490

**Published:** 2026-02-06

## Abstract

First Person is a series of interviews with the first authors of a selection of papers published in Biology Open, helping researchers promote themselves alongside their papers. Iris Sanou and Mathangi Lakshmipathi are co-first authors on ‘ Temporal dynamics of Sertoli and germ cell development in human foetal and prepubertal testis’, published in BIO. Iris is a PhD student in the lab of Dr Callista L. Mulder at Amsterdam UMC, Amsterdam, The Netherlands, and Professor Dr Rod Mitchell at The University of Edinburgh, Edinburgh, UK, investigating guiding cryopreserved testicular tissue through *in vitro* maturation and spermatogenic differentiation to restore male fertility and develop new strategies for the treatment of male infertility. Mathangi is a PhD student in the lab of Dr Callista L. Mulder at Amsterdam UMC, focusing on understanding and recreating the human testicular somatic niche *in vitro* to advance fertility preservation and male reproductive health.


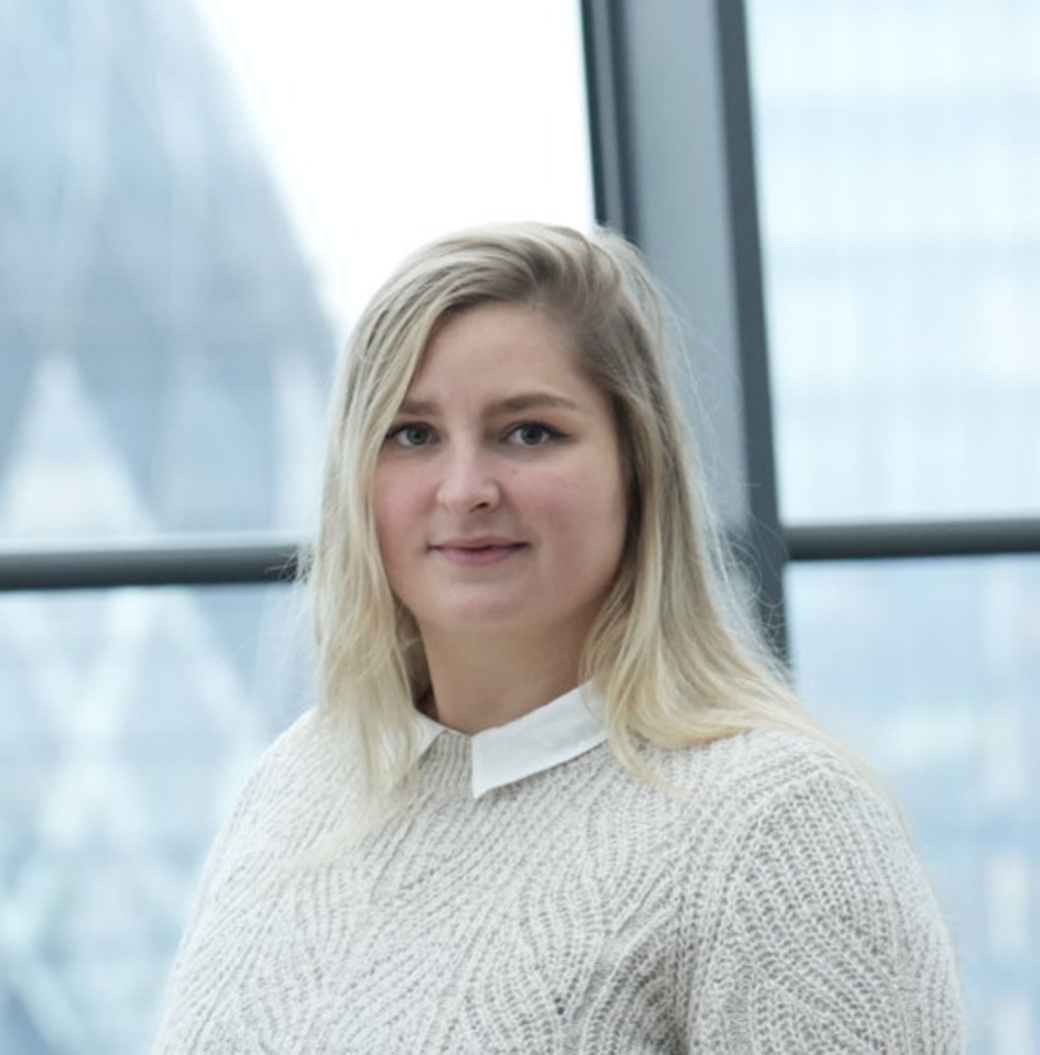


**Iris Sanou**

**Describe your scientific journey and your current research focus**

**I.S.:** My scientific journey started as a technician in a clinical pathology and cytology laboratory, where I was introduced to the fascinating microscopic world of human cells and the medical aspects of infertility. This experience led to a growing interest in reproductive health and the underlying biological mechanisms involved in this disease.

During my Bachelor's (Life Sciences and Chemistry), during which I completed an internship in a clinical fertility clinic, where I became particularly fascinated by spermatozoa. The idea that such a small and seemingly simple cell carries the capacity to create new life deeply intrigued me. During my internship in the fertility laboratory, I experienced how laboratory diagnostics can have a direct and meaningful impact on patients and their lives. Although I greatly valued my work in clinical diagnostics, those experiences motivated me to pursue a deeper scientific understanding of spermatogenesis at the molecular level. I therefore completed my Master's in Biomolecular Science, with a special focus on Developmental and Reproductive Biology.

Starting a PhD trajectory in my field of interest has allowed my clinical background and scientific training to integrate into a unified scientific approach. My research is conducted both in Amsterdam (Dr Callista Mulder, Professor Dr Ans van Pelt) and in Edinburgh (Professor Dr Rod Mitchell). These combined experiences have shaped my current research focus on male fertility, with an emphasis on spermatogonial stem cells and *in vitro* spermatogenesis. My research is driven by the clinical need for safe and effective fertility preservation strategies. While biobanking of testicular tissue is now offered as a fertility preservation option, the translation of spermatogonial stem cell-based therapies into clinical practice remains a major challenge. I am keen to continue to contribute to this matter.

**M.L.:** My fascination with the human body and how it functions was sparked at a very young age, encouraged by my parents through children's science books that explained organ systems and diseases, effectively training me for a lifetime of asking “but why?”. This early curiosity gradually grew into a desire to understand life at a deeper level, which led me to pursue a Bachelor's degree in Biotechnology at SRM University, India. With a strong academic record, I had the opportunity to carry out my Bachelor's thesis at the Immuno-engineering Research Laboratory of the Harvard–MIT-Health Sciences and Technology programme, where I was introduced to cancer biology and immunology and gained my first hands-on experience with research at an international level.

With a desire to explore biological problems at their roots, at the level of cells and their interactions, I went on to complete a Master's degree in Molecular Life Sciences with a major in Biomedical Research at Wageningen University and Research. Working across multiple laboratories during my Master's allowed me to broaden my scientific perspective, strengthen my critical thinking skills and explore diverse areas of biology.

I am currently a PhD candidate in the Reproductive Biology Laboratory at Amsterdam UMC, where my research focuses on developing a human induced pluripotent stem cell (hiPSC)-based culture system to model human *in vitro* spermatogenesis. My supervisory team is formed by Professor Dr Ans van Pelt, Dr Geert Hamer, and Dr Callista Mulder (PI on this project). In particular, I study the development and function of the testicular somatic niche, with an emphasis on Sertoli cells. By recreating key aspects of this niche *in vitro*, my work aims to improve our understanding of gonadal development to advance male reproductive health.

**Who or what inspired you to become a scientist?**

**I.S.:** From a young age, I was driven by curiosity and a strong desire to understand how things work. As a child, I spent hours experimenting with flowers, soap and water, trying to create my own mixtures. Looking back, these playful experiments were my first encounters with observation and discovery. The support of my parents and grandparents played an important role in shaping both my curiosity and creativity. Their encouragement allowed me to explore, experiment and develop my interests with confidence. Later, the guidance and encouragement of inspiring supervisors Ans van Pelt, Rod Mitchell and Callista Mulder helped shape my curiosity into a clear academic path and a career in scientific research.

**M.L.:** I've loved spending time in the lab ever since high school – lab days were always my favourite. I still remember the first time I passaged cells during my bachelor's and couldn't resist checking on them every day, thinking, “Look at me, I'm a scientist!”. Little did I know, my obsession with tiny cells would one day lead me to spend most of my days staring at them in different forms under a microscope and loving every minute of it. What makes it even more rewarding is knowing that all this curiosity and attention to detail could one day contribute to discoveries that make a real difference in people's lives.

**How would you explain the main finding of your paper?**

In our study, we mapped how cells in the human testis mature from early foetal life through childhood. It is generally believed that the testis starts preparing for sperm production just prior to the onset of puberty. We found that the cells supporting sperm formation and maturation, Sertoli cells, are most active in early childhood, before slowing down around age 6 as the testis begins to mature. Meanwhile, the cells that eventually become sperm divide at a slow and steady pace long before puberty. This timeline helps us delineate how the testis develops normally and provides a foundation for understanding the cellular changes that support sperm formation.

These models […] could eventually guide strategies to preserve or restore fertility in young boys requiring gonadotoxic treatments or in men facing infertility

**What are the potential implications of this finding for your field of research?**

Knowing this timeline is important because it gives researchers a reference for normal testis development, which can be used to build better laboratory models of sperm formation, like *in vitro* spermatogenesis. This has important implications for how we study, protect and model male fertility. These models can help us study gonadal development and male reproductive health more accurately and could eventually guide strategies to preserve or restore fertility in young boys requiring gonadotoxic treatments or in men facing infertility.


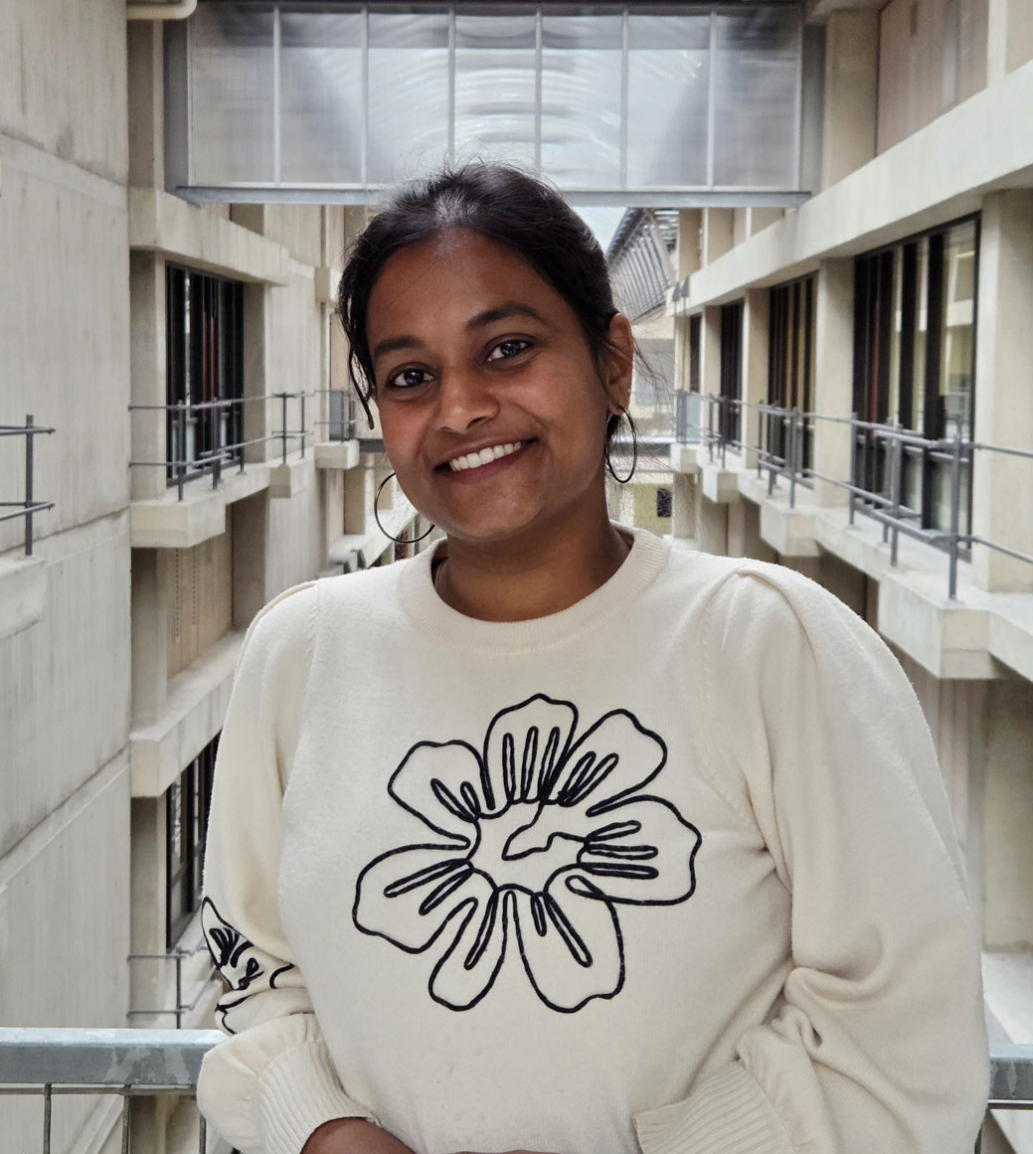


**Mathangi Lakshmipathi**

**Figure:**
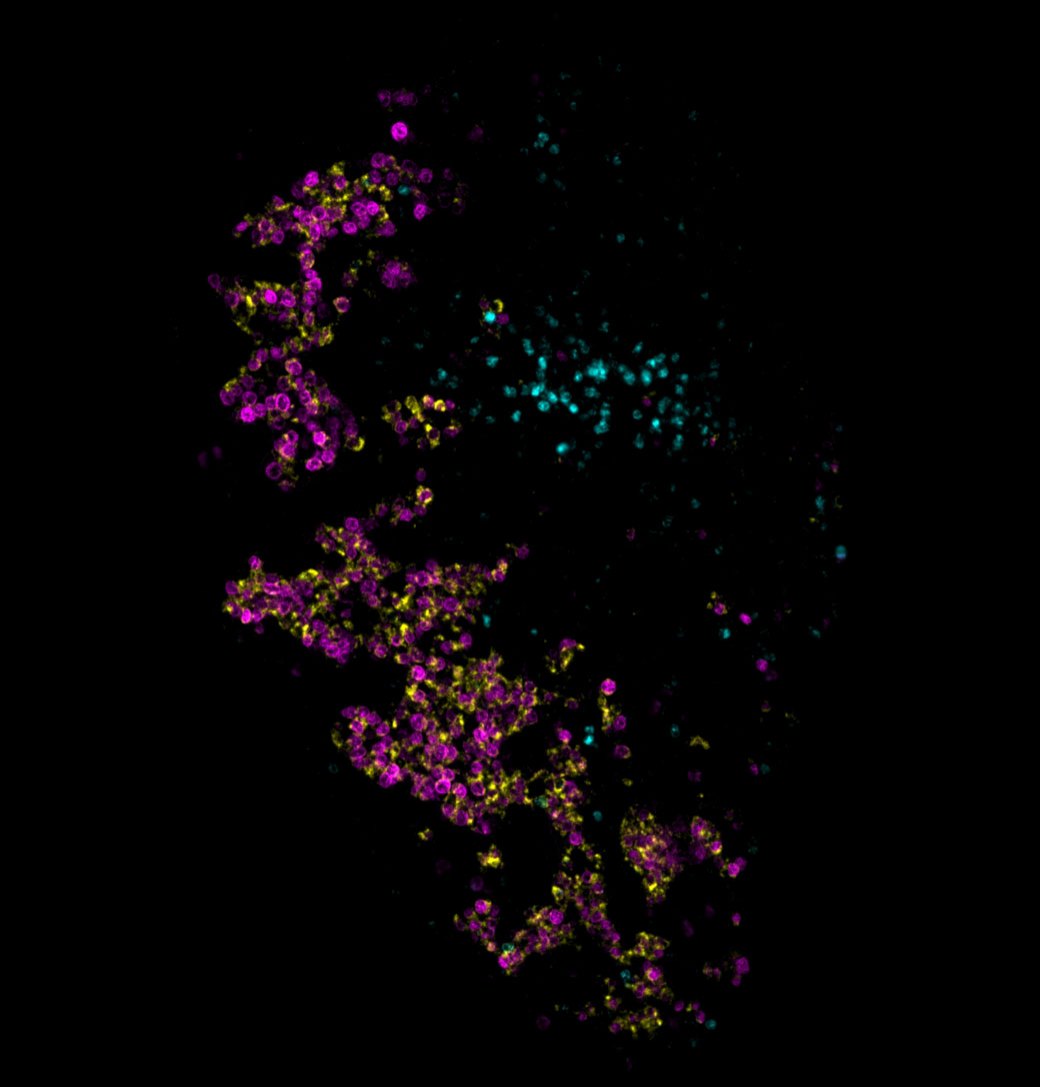
Immunofluorescence of the human foetal testis at post conception week 7, highlighting early Sertoli cell establishment (SOX9 in magenta, AMH in yellow and AR in cyan).

**Which part of this research project was the most rewarding?**

**I.S.:** One of the most rewarding aspects of this project was the collaboration with the team in Edinburgh. Through close contact with Rod Mitchell, I had the opportunity to visit their laboratory and to work alongside their team, and personally collect the specimens and create the unique sample collection that formed the basis of this study. Another highlight was sharing our work with the scientific community. Presenting our findings at conferences through talks and poster sessions, and being selected for an oral presentation at the European Testis Workshop 2025 for this project, was a great honour. It was exciting to see how much interest our work generated and how often the microscopic images in particular captured the audience attention. Engaging in discussions with other researchers and seeing genuine enthusiasm for our findings made the long hours in the laboratory feel truly worthwhile. Knowing that our work may serve as a reference for future research and applications in the field has been one of the most fulfilling aspects of this project.

**M.L.:** The most rewarding part of this project was unblinding our samples after the analysis and seeing all the pieces come together. It was incredibly satisfying to watch the story emerge from the data. And of course, generating all the beautiful, frame-worthy images from the study was a constant source of awe and excitement! Additionally, being invited to present this work as a speaker at the Dutch Society for Stem Cell Research (DSSCR) was a truly special and validating experience.

**What do you enjoy most about being an early-career researcher?**

**I.S.:** To me, being a researcher is the perfect blend of creativity, problem solving, social interaction and collaboration. It allows me to be curious, analytical and inventive at the same time, while working together with others toward a shared goal. I enjoy the freedom to explore ideas, develop new approaches and continuously create opportunities to learn. The process of turning a research question into an experiment, and an experiment into new insight, is deeply rewarding and keeps me motivated.

I greatly enjoy sharing my work with other researchers at conferences and scientific meetings. Presenting our results, discussing ideas and exchanging perspectives with colleagues from different backgrounds is inspiring and helps broaden my view on my own research.

**M.L.:** What I enjoy most about being an early-career researcher is the freedom and flexibility to explore new ideas and ask questions that haven't been answered yet. There's also the freedom to make mistakes and learn from them, which is an incredible part of the journey. I love the excitement when an experiment finally works, and even better, the new questions that arise from each result keep the work constantly stimulating. Even with frustrations and failed attempts, there's almost never a boring day in the lab. I also enjoy the fun of collaborating with other early-career researchers and slowly piecing together the full story from all our little puzzle pieces, which makes this career feel endlessly rewarding.

**What piece of advice would you give to the next generation of researchers?**

**I.S.:** Research is built on trial and error, and failure is not a sign that you are doing something wrong. It is often a sign that you are doing something new. For every experiment that works, many will not, and that is simply part of the journey. Do not struggle in silence. Share your challenges and bottlenecks with colleagues, supervisors and peers. Very often, others have faced the same problems, and progress happens faster when knowledge and experience are exchanged. Most importantly, stay curious and persistent. The long days in the laboratory, the repeated troubleshooting and the moments of doubt are all part of building something meaningful. With time and dedication your efforts will be rewarded.

**M.L.:** Much of the advice I would offer the next generation of researchers is advice I have received myself along the way. One importance lesson is learning to be comfortable with being bad at things; this gives you the freedom to take risks, try new approaches and step outside your comfort zone. Setbacks are an inherent part of science and does not reflect your ability as a researcher; once you learn how to bounce back, failures become far less paralysing. Equally important is celebrating even the smallest wins, whether it is a successful experiment, overcoming a writing block, or finally submitting a manuscript. Above all, kindness – to colleagues, collaborators and yourself – goes a long way in science.

Research is built on trial and error, and failure is not a sign that you are doing something wrong

**What's next for you?**

**I.S.:** My focus is preparing for my upcoming PhD defence in September this year. After this, I am eager to continue building a career in fertility and reproductive biology research, where I can further develop both my scientific and translational expertise. With my background in reproductive biology, biomolecular sciences and clinical laboratory work, I am looking for a position that allows me to combine fundamental research with clinical relevance. I am particularly interested in roles where I can contribute to advancing fertility preservation, male reproductive health and translational applications that directly impact patients.

**M.L.:** I am in the final year of my PhD and focused on successfully finalising my thesis. I am currently developing a hiPSC-derived model of the testicular niche, and I'm excited to see both how the project and I evolve along the way. As I approach the end of my PhD, I am seeking postdoctoral opportunities, and, in the long term, I hope my research can play a small, yet meaningful part in the bigger picture, contributing to work that makes an impact in people's lives.
